# Adrenal Insufficiency in Children With Nephrotic Syndrome on Corticosteroid Treatment

**DOI:** 10.3389/fped.2020.00164

**Published:** 2020-04-15

**Authors:** Karmila Abu Bakar, Khairunnisa Khalil, Yam Ngo Lim, Yok Chin Yap, Mirunalini Appadurai, Sangeet Sidhu, Chee Sing Lai, Azriyanti Anuar Zaini, Nurshadia Samingan, Muhammad Yazid Jalaludin

**Affiliations:** ^1^Pediatric Unit, Faculty of Medicine, University of Malaya, Kuala Lumpur, Malaysia; ^2^Pediatric Institute of the Hospital, Kuala Lumpur, Malaysia; ^3^Paediatric Nephrology Unit, Institute of Paediatrics, Kuala Lumpur, Malaysia

**Keywords:** adrenal insufficiency, steroid withdrawal, nephrotic syndrome, HPA axis, cortisol, low-dose Synacthen test, adrenal suppression

## Abstract

**Background:** Adrenal insufficiency can result from impaired functions at all levels of hypothalamic-pituitary-adrenal (HPA) axis. We here studied risk factors associated with adrenal insufficiency in children receiving prolonged exogenous steroid treatment for nephrotic syndrome.

**Method:**We performed low-dose Synacthen tests (LDSTs, 0.5 μg/m^2^) in children with steroid-sensitive nephrotic syndrome 4–6 weeks after discontinuation of the corticosteroid therapy. We measured early morning serum cortisol levels at baseline and at intervals of 10, 20, 30, and 60 min following the stimulation test. We defined normal HPA axis stimulation responses as those with peak cortisol cut-off values >550 nmol/L.

**Result:**We enrolled 37 children for this study research. All children enrolled had normal early morning cortisol levels. However, 13 (35.1%) demonstrated HPA axis suppression (by LDST) 4–+6 weeks after discontinuation of oral prednisolone. Nephrotic syndrome diagnosed before 5 years of age (OR, 0.75; 95% CI, 0.57–0.99; *p* = 0.043), and steroid-dependence [OR, 5.58; 95% confidence interval (CI), 1.06–29.34; *p* = 0.042] were associated with increased risk of developing adrenal suppression after steroid discontinuation.

**Conclusion:**HPA axis suppression, may go unnoticed without proper screening. A normal early morning cortisol level (275–555 nmol/L) does not exclude adrenal insufficiency in children with steroid-sensitive nephrotic syndrome. Further screening with LDSTs, particularly in children younger than 5 years at diagnosis, may be warranted.

## Introduction

The most common glomerular disease in childhood is idiopathic nephrotic syndrome (NS), for which corticosteroids are the first line of treatment. Almost 80% of children with NS demonstrate steroid responsiveness, achieving complete remission within 4 weeks. However, ~40–50% of these children are steroid dependent and require a long course of steroids, leaving them vulnerable to the adverse effects of chronic steroid usage, such as stunted growth, hypertension, obesity, secondary diabetes, vitamin D deficiency and secondary osteoporosis, cardiomyopathy, and hypothalamic-pituitary-adrenal (HPA) axis suppression. Most of these conditions are well known, but HPA axis suppression has to date been understudied and under-reported.

The recommended treatment regimen for NS at diagnosis, according to the KDIGO guidelines (1), is with induction of prednisolone at 60 mg/m^2^/day, or 2 mg/kg/day, (maximum 60 mg/day) for 4–6 weeks. This will be followed by alternate-day prednisolone given in single doses at 40 mg/m^2^ or 1.5 mg/kg (maximum 40 mg on alternate days), for 2–5 months with tapering of the dose. This is a longer duration of steroid therapy compared to the original ISKDC guideline (2) which consisted of 4 weeks induction and 4 weeks of 40 mg/m^2^ 3 days a week. Recently, PREDNOS trial (3) compared the pattern of disease relapse in patients receiving 8 weeks vs. extended 16 weeks of corticosteroids. There was no significant difference in time to first relapse in these two groups. They found that extended corticosteroid treatment reduced the healthcare resource use and only made a small improvement in quality of life. Treatment of relapse is different from initial presentation. Alternate day dosing of 0.1–1 mg/kg for at least 3 months is recommended in frequently relapsing and steroid dependent NS. The steroid weaning regimen may vary based on the individual patient's previous experiences with relapses, steroid dependency, frequency of relapses, steroid toxicity and the use of steroid sparing agent. Close monitoring is imperative to detect complications associated with prolonged use of high-dose corticosteroids.

The physiological secretory rate of endogenous steroids in the intact HPA axis was ~6 mg/m^2^/day. Exogenous steroid doses were adjusted above this secretory rate (8–10 mg/m^2^/day) as their bioavailability is reduced by gastric acids and first-pass metabolism in the liver. High-dose steroids are defined as supraphysiological steroid levels. HPA axis suppression is dependent on the total duration, total cumulative dose, and potency of the steroid used (4).

Adrenal insufficiency can be primary, secondary, or tertiary. Primary adrenal insufficiency results from intrinsic adrenal cortex diseases. Secondary and tertiary adrenal insufficiencies are caused by impaired production or action of corticotrophin, and are collectively known as central adrenal insufficiency. Overall, the prevalence of secondary adrenal insufficiency is 150–280 per million inhabitants in a population (5) and the condition can occur in children on high-dose and prolonged steroid prescriptions (4).

Various stimuli may activate the HPA axis to maintain normal body homeostasis and to combat stress, such as that induced by infections and endotoxins (6),absence or reduced glucocorticoid negative feedback (7), and hypoglycemia. Suppression of this critical mechanism via endogenous or exogenous pathways may alter the body's adaptive processes, triggering a pathogenic cascade. Adrenal insufficiency is associated with significant morbidity and mortality when inadequately treated during periods of intercurrent illness (8).

Different methods of assessing the HPA axis function exist. The insulin tolerance test and the Standard Short Synacthen test (SSST) were frequently used, but their limited reliability, tediousness, and associated adverse events led to the low-dose Synacthen test (LDST) being the preferred test nowadays. Moreover, LDST can detect mild degrees of adrenal insufficiency missed by SSST (9).

## Materials and Methods

### Study Population

**Figure 1:**
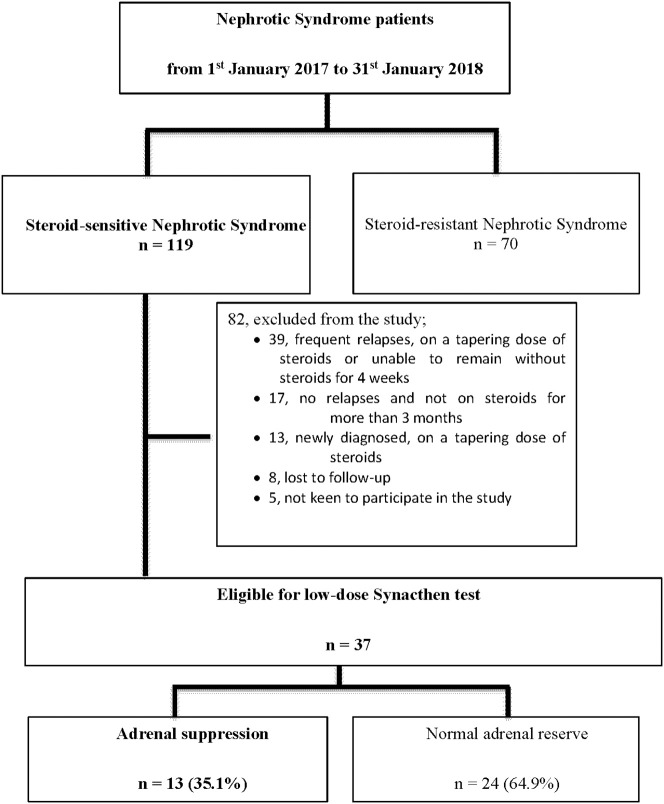
Overview of the study flow.

We identified 119 patients from the admissions list of the Pediatrics Institute of the Hospital Kuala Lumpur (Malaysia) between January 2017 and January 2018. We identified 119 patients as having steroid-sensitive NS, and enrolled 37 patients who fit the study inclusion criteria. Eligible patients had: (i) steroid-sensitive idiopathic NS; (ii) achieved remission; and (iii) been off steroids for 4–6 weeks. We excluded patients with: (i) steroid-resistant NS; or (ii) secondary NS. Figure 1 summarizes the selection process (Table 1).

**Table 1:** Common definitions in nephrotic syndrome.

Remission	Urine albumin nil or trace for three consecutive days
Relapse	Urine albumin 3+ or more for three consecutive days
Frequent relapses	Two or more relapses in the initial 6-month period or more than three relapses in any 12 months
Steroid dependence	Two consecutive relapses when on alternate day steroid therapy or within 14 days of its discontinuation
Steroid resistance	Absence of remission despite therapy with daily prednisolone at a dose of 60 mg/m^2^/day

### Data Collection

We used a pre-defined standardized data collection sheet for extracting data that contained information on patients' socio-demographic information (gender, age, ethnicity), anthropometric measurements, age at diagnosis, total number of relapses, and total duration of steroid use before LDST.

We collected blood samples for morning serum cortisol and ACTH levels, and for the LDST after steroid discontinuation during a planned visit. We then examined the patients every 3 months for a year to monitor their disease progress, their growth parameters, and to detect relapses or steroid toxicity.

### LDST

Synacthen was given intravenously (0.5 μg/m^2^) and serum cortisol levels were measured before and after the administration. The peak cortisol response at any time within 60 min should exceed 550 nmol/L (10). We used a lower Synacthen dose 0.5 μg/m^2^ than the conventional 250 μg/m^2^ dose based on the observation that the low-dose ACTH stimulation test using 1 μg of tetracosactrin can be used to detect mild secondary adrenal insufficiency (9). A cortisol increment at 30 min above the basal level is a measure of adrenal reserve and a low absolute level indicates adrenal insufficiency.

We educated parents about hydrocortisone replacement at times of illness or stress during transient adrenal insufficiency periods (11, 12). We informed parents to give oral hydrocortisone at three times the physiological replacement dose (30 mg/m^2^/day) divided into three equal doses) during moderate illnesses such as fever and minor injury (this is called hydrocortisone “stress dosing”). During severe illnesses such as serious injury or trauma, or for anesthesia for surgery, we prescribed intravenous hydrocortisone at 10 times the physiological replacement dose (100 mg/m^2^/day divided into four equal doses) (11).

For study subjects who relapsed during the study period, treatment was instituted as per International Study of Kidney Disease in Children (ISKDC) guideline. Patients who developed an intercurrent illness during the study period were treated as per other children with NS. Assessment of the volume status, the need for antibiotics and steroid replacement therapy was on the discretion of the treating physician.

### Ethical Considerations

We registered this study with the National Medical Research Register (NMRR) under the identification number NMRR-16-1427-30586 (IIR). The Medical Research Ethics Committee (MREC), the University Malaya Medical Centre (UMMC) approved the study under the registration number MREC UMMC ID: 2016816-4142. This study was supported by a grant from the University Malaya Research Fund Assistance (BKP034-2015). We obtained written informed consent from all parents or guardians of the patients prior to starting the study. All children enrolled were also required to sign an assent form or to agree verbally. We performed all tests in accordance with approved guidelines.

### Statistical Analysis

We used descriptive statistics to compare baseline characteristics between children with adrenal suppression and those with normal adrenal function. We expressed numerical data as means and standard deviations, and tabulated categorical variables as predicted numbers and percentages. We first used simple logistic regression analyses to determine unadjusted associations of factors of interest, and then applied multivariate analyses using the logistic regression model to identify predictors of HPA insufficiency. We performed data analyses using the Statistical Package for Social Science software version 23.0. The data that support the findings of this study are available upon request from the corresponding author. The data are not publicly available due to privacy or ethical restrictions.

## Results

Between 1st January 2017 and 31st January 2018, a total of 189 patients received treatment for NS in the Pediatric Institute of the Hospital Kuala Lumpur in Malaysia. Of 189 patients, 70 (37%) were steroid-resistant and 119 patients were steroid-sensitive, but only 31% of the patients (37 of 119) fit the eligibility criteria for enrolment; we excluded 69 patients, lost eight to follow-up, and encountered five patients who chose not to participate in the study (Figure 1).

Our study population showed a greater preponderance of males, with a male to female ratio of 3.6:1. The majority of patients were of Malay ethnicity and consisted of 26 patients (70.3%) and the rest were Chinese and Indians (in almost equal proportion). The mean age at diagnosis of NS was 3.38 years (±2.28), and the mean age at LDST was 9.47 years (±3.73). Thirty-four out of 37 patients (91.9%) were 5 years old or younger. The mean duration of the NS was 6.09 years (±3.44), with 21/37 (43.2%) having the illness for more than 5 years (Table 2).

**Table 2:** Demographic and clinical characteristics of patients with normal HPA axis and HPA axis suppression.

**Characteristics**		**Normal HPA axis (*n* = 24) (64.9%)**	**HPA axis suppression (*n* = 13) (35.1%)**
Gender	Male	19 (79.2)	10 (76.9)
	Female	5 (20.8)	3 (23.1)
Race	Malay	16 (66.7)	10 (76.9)
	Chinese	5 (20.8)	1 (7.7)
	Indian	3 (12.5)	2 (15.4)
Age at diagnosis, years, mean ± SD^*^		3.83 ± 2.69	2.54 ± 0.80
Age at diagnosis	0–5 years	21 (87.5)	13 (100)
	6–11 years	2 (8.3)	0 (0.0)
	12–18 years	1 (4.2)	0 (0.0)
Age at LDST, years, mean ± SD^*^		10.44 ± 4.25	7.67 ± 1.32
Age at LDST	0–5 years	5 (20.8)	2 (15.4)
	6–11 years	10 (41.7)	11 (84.6)
	12–18 years	9 (37.5)	0 (0.0)
Duration of illness, years, mean ± SD^*^		6.61 ± 3.99	5.13 ± 1.86
Duration of illness	≤ 5 years	10 (41.7)	6 (46.2)
	> 5 years	14 (58.3)	7 (53.8)
Duration of steroid use, weeks, mean ± SD^*^		30.54 ± 35.31	66.05 ± 121.25
Duration of steroid use	<20 weeks	13 (54.2)	7 (53.8)
	≥ 20 weeks	11 (45.8)	6 (46.2)
The dose of steroid use, mg/m^2^/days, mean (SD)^*^		22.37 ± 8.30	25.63 ± 11.36
Steroid-dependent	No	14 (58.3)	3 (23.1)
	Yes	10 (41.7)	10 (76.9)
Frequent relapse	No	20 (83.3)	9 (69.2)
	Yes	4 (16.7)	4 (30.8)
Steroid toxic	No	8 (33.3)	6 (46.2)
	Yes	16 (66.7)	7 (53.8)
Use of steroid-sparing agent (SSA)	No	9 (37.5)	8 (61.5)
	Yes	15 (62.5)	5 (38.5)
Immuno-suppression prior to LDST	Prednisolone alone	19 (79.2)	11 (84.6)
	Combination with SSA	5 (20.8)	2 (15.4)
Comorbid diseases	No	17 (70.8)	11 (84.6)
	Yes	7 (29.2)	2 (15.4)
ACTH, pmol/L, mean ± SD		20.52 ± 14.20	40.92 ± 85.14
ACTH Result	Normal	22 (91.7)	10 (76.9)
	Abnormal	2 (8.3)	3 (23.1)

* *SD, standard deviation*.

Almost two thirds of the patients, 62.2% (23/37), had evidence of steroid toxicity. However, only 54% (20/37) had received a steroid-sparing agent (SSA). We possess no data that can explain this discrepancy. The SSAs prescribed were cyclophosphamide (13), levamisole (12), cyclosporine (7), rituximab (3), mycophenolic acid (1), and tacrolimus (1). Four patients received three or more types of SSA, six patients received two types of SSA, and the remaining 10 patients received a single agent. We categorized the steroid toxicities according to the affected system: immunosuppression; cardiovascular (hypertension); eye involvement (cataract, glaucoma); and musculoskeletal (short stature, cushingoid features, and skin striae). Eight out of 37 patients (21.6%) had frequent relapses and 20 (54.1%) patients were steroid dependent.

Thirteen out of 37 patients (35.1%) had HPA axis suppression. All of them were younger than 5 years of age and 76.9% were boys (*n* = 10/13). The mean durations of steroid usage were 66 (±121.25) weeks in those who developed HPA axis suppression and 30 (±35.31) weeks in those who did not. Ten of those patients with HPA axis suppression (76.9%) were steroid dependent.

We found that children with a younger age (mean age at diagnosis of 2.54 years) were more likely to develop HPA axis suppression than older children (95% CI, 0.567–0.990). Steroid-dependent patients were six times more likely to develop HPA axis suppression than to maintain normal HPA axis function (95% CI, 1.06–29.34) (Table 3).

**Table 3:** Multivariate analysis for children with nephrotic syndrome on corticosteroid treatment.

**Variables**		ß^*^	Df^**^	***p*-value**	**Adjusted ^**+**^OR (95% ^**++**^CI)**
Age at diagnosis		−0.288	1	0.043	0.749 (0.567–0.990)
Steroid-dependent	No	1.719	1	0.042	5.58 (1.06–29.34)
	Yes				

^*^ß, beta; ^**^Df, degrees of freedom; ^+^OR, odd ratio; ^++^CI, confidence interval; LDST, low-dose Synacthe n test.

**Figure 2:**
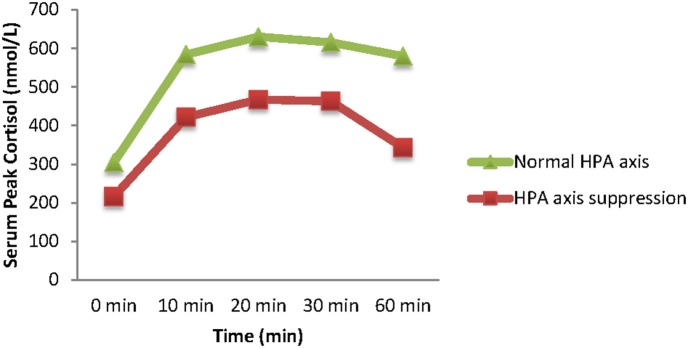
Serum peak cortisol concentrations (means) from baseline (time 0) following stimulation with 0.5 μg/m^2^ Synacthen (10, 20, 30, and 60 timepoints).

The mean basal plasma cortisol concentration was significantly higher by 40% in patients with a normal adrenal response than in patients with a suppressed adrenal response [303.92 (±133.99) vs. 215.69 (±102.61) nmol/L, *p* = 0.012]. After the injection of 0.5 μg/m^2^ Synacthen, both groups of patients demonstrated serum cortisol peaks at 20 min. The mean 20-min peak serum cortisol concentration was lower in the children with suppressed responses than in those with normal responses; this difference, however, was not statistically significant [467.54 (±69.16) and 630.33 (±97.86), respectively, *p* = 0.869] (Figure 2).

## Discussion

We analyzed cortisol levels in patients with steroid-sensitive NS using LDST and confirmed that 13 of them (35.1%) had HPA axis suppression. Multivariate analyses showed that younger age at diagnosis and steroid dependency were the two significant factors associated with HPA axis suppression. We defined HPA axis suppression as cortisol levels <550 nmol/L despite stimulation. This definition may help to anticipate adverse outcomes associated with inappropriate cortisol responses during “stress periods” in children on steroid therapy.

The duration of steroid usage in children with NS varies with the frequency of relapses, steroid dependency, presence of steroid toxicity, and different medical practices. In our center, we administer steroids as per the ISKDC guideline. Case-to-case evaluations are frequent as some patients maintain remission states and avoid relapses when given prolonged steroid courses.

The HPA axis functions as a complex neuroendocrine feedback mechanism crucial during stress adaptation (14); it is influenced by circadian and ultradian rhythms (15). Animal studies have shown that the HPA axis regulates the secretion of glucocorticoids, which have dominant effects in regulating cardiovascular, metabolic, cognitive, and immunological states (13, 16, 17). The paraventricular nuclei (PVN) of the hypothalamus initiate axis activation (18, 19). Exposure to stressors stimulates the secretion of corticotrophin-releasing hormone (CRH), and this hormone eventually stimulates the pituitary gland to secrete adrenocorticotropic hormone (ACTH). ACTH in turn binds to its receptors on the adrenal gland and induces cortisol secretion (20, 21), which inhibits ACTH and CRH production (negative feedback mechanism), in a manner similar to that in children with excess exogenous glucocorticoids (22).

Children with NS receive supraphysiologic doses of steroids that may potentially inhibit cortisol production. When this inhibition lasts longer than the duration of the corticosteroid exposure, it is called adrenal suppression. Although the effect may be transient, it can result in significant morbidity during periods of physiologic stress (23). In children receiving high-dose steroids, adrenal suppression is not apparent and clinicians need to be aware that abrupt withdrawal may trigger adrenal crises (4, 22).

The suppression of the HPA axis can occur after a single dose of steroid, but it typically recovers quickly. However, the long-term use of systemic corticosteroids may take a long time to recover (24). Studies have looked at the effects of corticosteroid use on the HPA axis and at the corresponding recovery times. The recovery time depends on the glucocorticoid potency and its types, the duration of therapy, and the weaning protocols; therefore, estimations are difficult, and comparisons between studies are complex also because of the different diagnostic tests used to assess the adrenal function.

In our study, the local prevalence of adrenal suppression in children with NS 4–6 weeks after steroid discontinuation was 35.1%. This figure is lower than that reported in a study by Abeyagunawardena et al. (25), which showed that 20 of 32 (62.5%) children on alternate-day prednisolone for steroid-dependent NS had HPA axis suppression. Furthermore, their cut-off cortisol level to diagnose adrenal suppression was found to be <500 nmol/L. Mantan et al. (26) also studied children on low dose steroids and 28 of 70 children had adrenal suppression. Diagnosis is based on a single early morning dose of serum cortisol. Those at a risk of acquiring adrenal suppression in his study were mainly frequent relapsers and steroid resistant NS.

The variety of investigations used and different cut-off values to examine the HPA axis are potential caveats concerning the validity of the assessment results. Recently, Mongioi et al. (27) studied the accuracy of LDST in 103 adults. All of them had primary AI or hypothalamic-pituitary diseases. Those receiving steroids were excluded. The receiver operating characteristic curve showed 100% sensitivity and 67.3% specificity when 500 nmol/L was used as the serum cortisol cut-off value. Our study, however, is based on the study done by Tordjman et al. (9), which used 550 nmol/L of serum cortisol as the cut-off value in LDST, resulting in 100% sensitivity and 89% specificity.

We found steroid toxicity in 62.2% of our cohort, suggesting that predicting which patients are at risk of developing adrenal crisis is impossible based on steroid toxicity alone. In a resource limited setting, conducting screening tests for HPA axis suppression in every patient on corticosteroid treatment is additionally unfeasible. Therefore, predicting patients who warrant screening for adrenal suppression is important. Based on our study findings, patients younger than 5 years, and who are steroid-dependent, should be screened for adrenal suppression by measuring both a morning serum cortisol level and an LDST. Identifying children at risk for adrenal suppression early would reduce the burden of illness in terms of morbidity, mortality, and cost of care.

We acknowledge the limitations of our study. We had no data on the frequency of hospital admissions, severity of intercurrent illnesses, or recovery rates to compare between patients with HPA axis suppression and those without HPA axis suppression. We also failed to examine the recovery periods in children with HPA axis suppression.

In conclusion, a life-threatening HPA axis suppression may go unnoticed without proper screening. A normal early morning cortisol level (275–555 nmol/L) does not rule out adrenal insufficiency in children with steroid-sensitive NS. Further screening with LDSTs may be warranted, particularly in children younger than 5 years of age at diagnosis.

## Data Availability Statement

The datasets analyzed in this article are not publicly available. Requests to access the datasets should be directed to Karmila Abu Bakar, karmila@um.edu.my.

## Ethics Statement

The studies involving human participants were reviewed and approved by NMRR. Written informed consent to participate in this study was provided by the participants' legal guardian/next of kin. Written informed consent was obtained from the individual(s), and minor(s)' legal guardian/next of kin, for the publication of any potentially identifiable images or data included in this article.

## Author Contributions

All authors worked together in designing the study, getting ethics approval, recruiting patients, performing the low-dose synacthen test, analyzing the data, and writing up the paper.

### Conflict of Interest

The authors declare that the research was conducted in the absence of any commercial or financial relationships that could be construed as a potential conflict of interest.
